# Case Report of a Novel EVC Gene Mutation in Ellis–van Creveld Syndrome: Implications for Pediatric Dental Management

**DOI:** 10.1155/crid/6628305

**Published:** 2025-12-19

**Authors:** Mahnegar Shariati, Sara Tavassoli-Hojjati, Amirahmad Pahlavan Hoseini, Hanieh Jadidi

**Affiliations:** ^1^ Pediatric Department, School of Dentistry, Iran University of Medical Sciences, Tehran, Iran, iums.ac.ir; ^2^ Department of Pediatric, Faculty of Dentistry, Tehran Medical Sciences, Islamic Azad University, Tehran, Iran, azad.ac.ir; ^3^ Department of Prosthodontics, Faculty of Dentistry, Tehran Medical Sciences, Islamic Azad University, Tehran, Iran, azad.ac.ir

**Keywords:** anodontia, chondroectodermal dysplasia, Ellis–van Creveld syndrome, heart defects, oral manifestations, polydactyly

## Abstract

**Introduction:**

Ellis–van Creveld (EVC) syndrome, also known as chondroectodermal dysplasia, is a rare autosomal recessive disorder that affects multiple embryonic tissues. It is primarily caused by mutations in the *EVC* gene.

**Patient Information:**

We report an 11‐year‐old male diagnosed with EVC syndrome, who carries a novel homozygous pathogenic mutation, c.1750delC (p.Q584Rfs∗4), in the *EVC* gene, identified through whole‐exome sequencing (WES) and confirmed by Sanger sequencing. Clinical features included multiple aberrant frenula, disproportionate short stature, polydactyly, and dystrophic nails.

**Dental Management:**

The patient received multidisciplinary dental care, including composite restorations and prosthodontic rehabilitation with partial dentures adapted for future tooth eruption. The identification of this specific *EVC* mutation informed anticipatory dental planning, leading to individualized management strategies for optimal dental care.

**Conclusions:**

This case highlights a novel *EVC* gene mutation, underscoring the importance of genetic analysis in guiding comprehensive pediatric dental care for EVC syndrome. Early recognition of dental manifestations, coupled with molecular confirmation, supports tailored interventions to optimize oral health outcomes.

## 1. Introduction

Ellis–van Creveld (EVC) syndrome (MIM #225500, ORPHA: 289), also known as chondroectodermal dysplasia, is a rare autosomal recessive congenital disorder, involving all three embryonic layers [1, 2]. The syndrome was first described by Simon van Creveld and Richard Ellis in 1940 [3, 4]. The exact prevalence of EVC is unknown and is estimated at approximately seven per million [5], with no gender predilection [1]. Higher prevalence rates have been reported in Amish, Brazilian, Ashkenazi Jewish, and Arab communities [6–8]. The majority of EVC cases are related to mutations in the EVC (MIM #604831) and EVC2 (MIM #607261) genes located on chromosome 4p16 [9, 10]. Clinical manifestations vary considerably but commonly include disproportionate short stature, particularly involving the limbs and ribs, postaxial polydactyly, dysplastic nails, and congenital heart defects, which occur in more than half of affected individuals [11–14]. Oral and dental abnormalities are frequent and may significantly affect function and aesthetics. These features include tooth agenesis, peg‐shaped teeth, taurodontism, natal or neonatal teeth, delayed eruption, microdontia or macrodontia, premature exfoliation, and enamel hypoplasia [3, 15–18]. Additional intraoral findings often observed in EVC include multiple aberrant frenula, lip ties, labiogingival adhesions, alveolar notching, serrated mandibular incisors, and submucosal or palatal clefts [5, 14, 18–21]. Given the clinical variability and the potential impact of dental anomalies on function, aesthetics, and quality of life, early recognition and multidisciplinary management—particularly from a pediatric dental perspective—are essential.

This case describes an 11‐year‐old male with a previously unreported *EVC* gene mutation. It highlights the dental challenges associated with EVC syndrome, emphasizing early diagnosis, genetic evaluation, and coordinated multidisciplinary care.

## 2. Patient Information

An 11‐year‐old male patient, without cognitive or motor impairment, was referred from a private clinic to the Department of Pediatric Dentistry at Islamic Azad University, Tehran, Iran, with delayed tooth eruption as the primary concern. The patient′s parents are first cousins, and the child was born of a consanguineous marriage in Torkaman, a province located in northern Iran.

No family history or phenotypic traits suggestive of EVC syndrome were reported. The patient′s parents and two siblings were in good general health, with no history of dental anomalies, delayed tooth eruption, malocclusion, or craniofacial abnormalities. There were no hereditary dental or craniofacial conditions noted in the extended family.

At birth on August 20, 2012, the patient weighed 2800 g (12th percentile), had a length of 36 cm (1st percentile), and a head circumference of 49 cm (99th percentile). These findings indicated disproportionate short stature with relative macrocephaly according to the CDC growth charts. At 12 years, there was mild growth in weight and height, maintaining the same percentiles. By 13 years, the patient showed evidence of early pubertal growth acceleration, with macrocephaly remaining relative to the stature (Table 1).

**Table 1:** Anthropometric measurements at birth and at ages 12 and 13 years, with percentile ranks based on CDC growth charts.

**Age**	**Weight (kg)/percentile (CDC)**	**Height (cm)/percentile (CDC)**	**Head circumference (cm)/percentile (CDC)**
Birth (Aug. 20, 2012)	2.8 12th	36 1st	49 99th
12.0 years (measured)	23.5 ≈5th	128.0 ≈5th	Not measured
13.0 years (measured)	29.5 5th–7th	138.2 ≈5th	54.4 ≈70th

The patient′s prenatal and delivery history were uneventful, with no exposure to radiation or medications during pregnancy. At 8 days old, he was diagnosed with newborn jaundice and underwent phototherapy for 2–3 days. Developmental milestones, including walking, talking, and acquiring literacy skills, were achieved within expected time frames. Vision and hearing test results were normal, with no abnormalities detected.

The patient′s medical history includes a congenital cardiac condition involving an extra vein, which was evaluated at 4 years of age and treated surgically. Additionally, the patient underwent surgical intervention to remove a postaxial finger and to correct bilateral genu valgum (knock knees). To date, the patient has undergone seven surgical procedures related to cardiac, digital, and orthopedic abnormalities.

## 3. Clinical Findings

During the physical examination, the patient presented with short stature and disproportionate body proportions. The lower limbs demonstrated deformities with dysplastic genu valgum (Figure 1a), findings consistent with acromesomelic short‐limb dwarfism.

Figure 1Clinical manifestations of the patient with Ellis–van Creveld syndrome. (a) Patient stature demonstrating disproportionate short stature with short extremities, lower limb deformities and dysplastic genu valgum (arrow). (b) Frontal facial view showing mild dolichocephaly, midface hypoplasia, short slightly upslanting palpebral fissures, and a broad nasal tip. (c) Lateral view confirming low‐set ears, normally formed antihelix, and midfacial retrusion. (d, e) Bilateral hand anomalies including brachydactyly, clinodactyly of the fifth fingers (arrow), postaxial polydactyly, and dystrophic nails.

**Figure:**
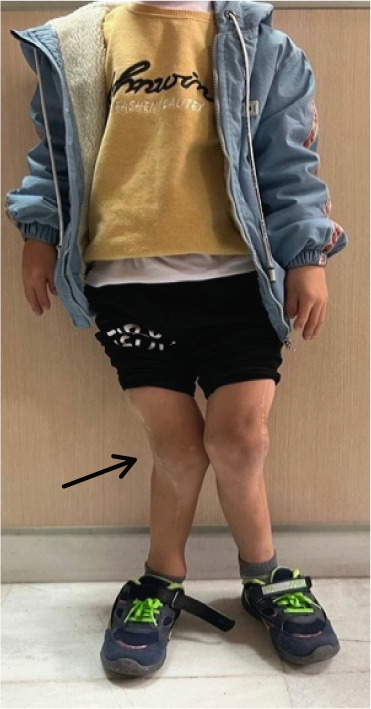
(a)

**Figure:**
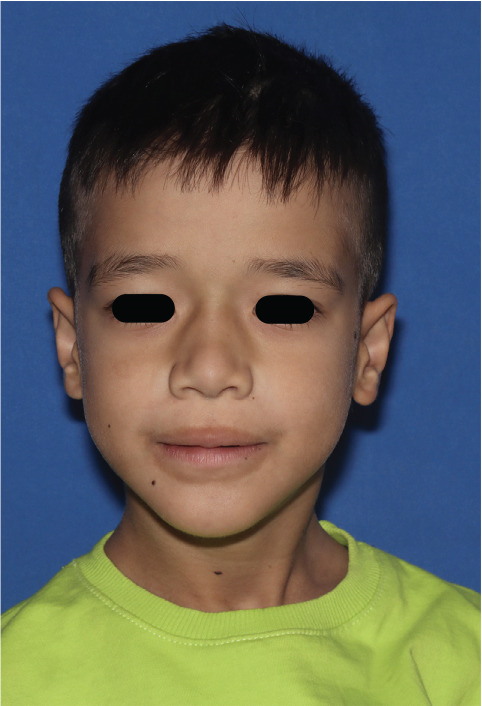
(b)

**Figure:**
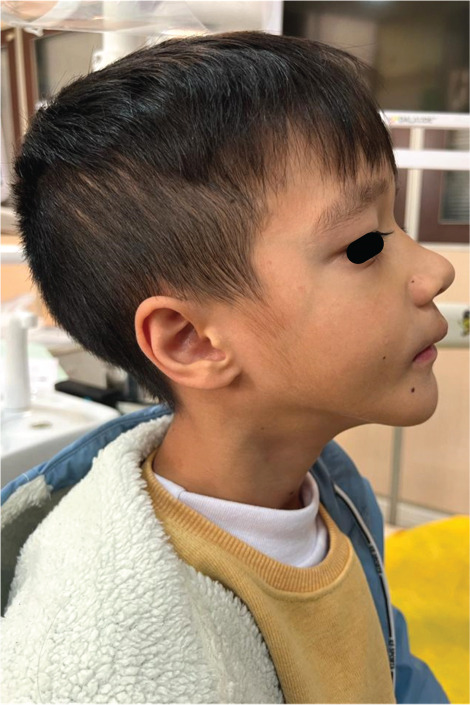
(c)

**Figure:**
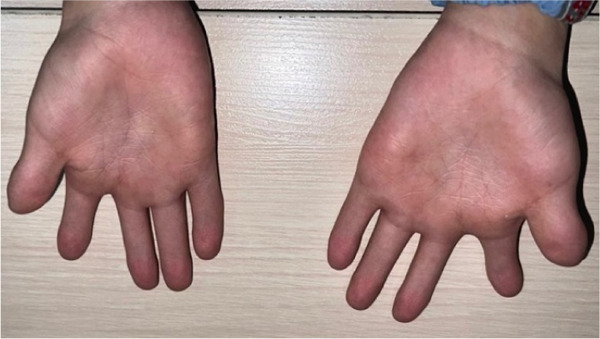
(d)

**Figure:**
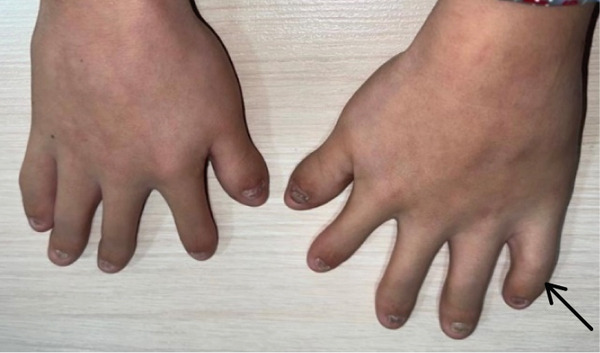
(e)

On facial examination, the patient exhibited mild dolichocephaly, midface hypoplasia, and low‐set ears. The palpebral fissures appeared short and slightly up slanting, and the nasal tip was broad (Figure 1b). The antihelix was normally formed without additional auricular anomalies (Figure 1c).

Examination of the extremities revealed dystrophic changes in both fingernails and toenails, as well as brachydactyly, clinodactyly of the fifth finger, and postaxial polydactyly present since birth. Despite these digital anomalies, finger and wrist mobility remained within normal limits (Figure 1d,e).

Intraoral examination (Figure 2) revealed congenital oligodontia, a finding further confirmed radiographically. The palatal vault was high‐arched, and multiple aberrant frenula were observed. Additionally, Maxillary Canines 13 and 23 showed talon cusp formation.

Figure 2Intraoral views of the patient. (a) Right buccal view, (b) frontal view, (c) left buccal view, (d) mandibular occlusal view, and (e) maxillary occlusal view. Findings include oligodontia, high‐arched palate, and multiple aberrant frenula (black arrows). Talon cusp formation is present on the maxillary canines (white arrows). Note the crown and root dysmorphisms affecting the remaining dentition.

**Figure:**
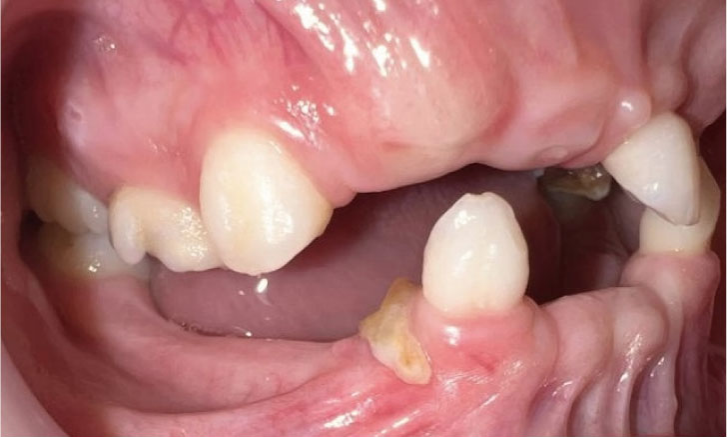
(a)

**Figure:**
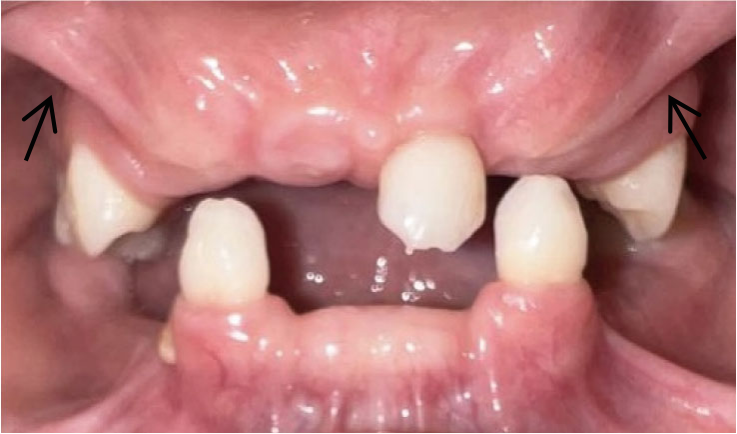
(b)

**Figure:**
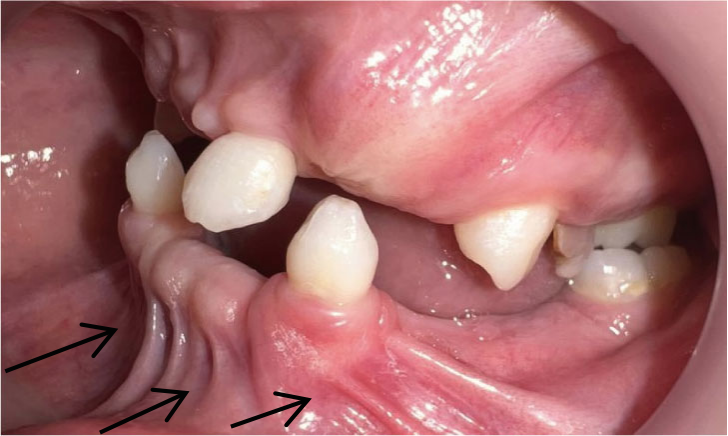
(c)

**Figure:**
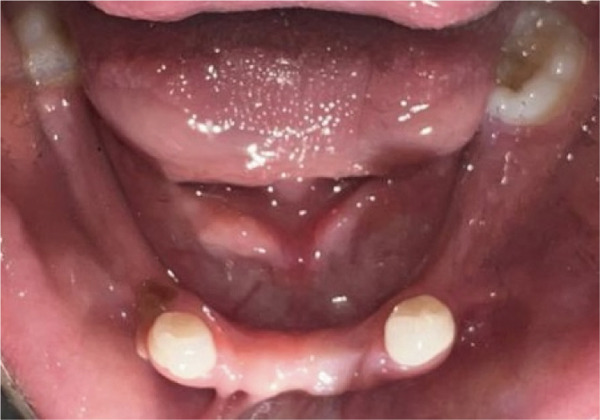
(d)

**Figure:**
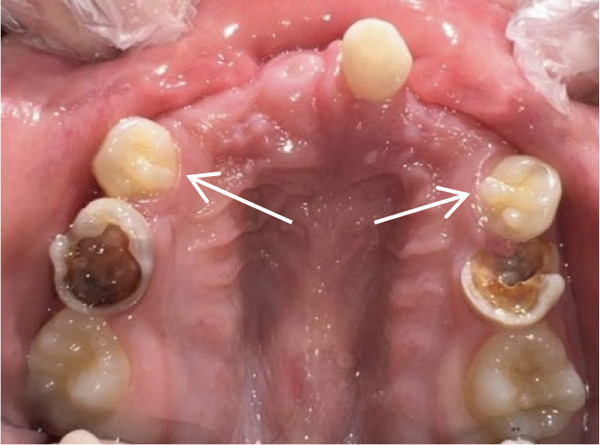
(e)

**Figure 3:**
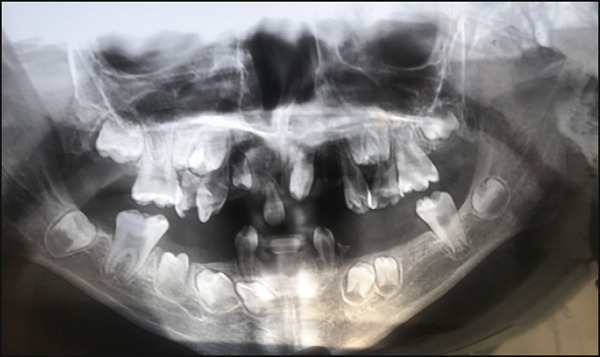
Panoramic radiograph demonstrating multiple dental anomalies. There is congenital absence of several Permanent Teeth #12, #21, #32, #31, #41, #42, and #43 and overretained Primary Teeth #61, #73, and #83. Note the crown and root dysmorphisms, shortened root morphology, and abnormal configuration of pulp chambers and root canals, all of which are characteristic of developmental disturbances associated with Ellis–van Creveld syndrome.

**Figure 4:**
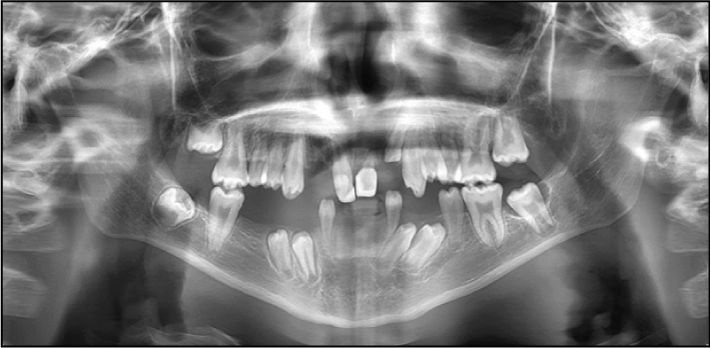
Panoramic radiograph at 2‐year follow‐up demonstrating persistent dental anomalies. Several teeth remain unerupted or impacted, including #17, a tooth in the area of #22, #27, #37, #34, #33, #44, #45, and #47. Additionally, overretained primary teeth #61, #73, and #83 are observed.

The panoramic radiograph (Figure 3) revealed multiple congenitally missing teeth, including #12, #31, #32, #33, #41, #42, and #43. Additionally, overretained Primary Tooth #6 was observed. At 13 years of age (Figure 4), several teeth remained unerupted or impacted, including #17, a tooth in the area of #22, #27, #37, #34, #33, #44, #45, and #47. Overretained Primary Teeth #83 and #84 were also present. Notably, the pattern of agenesis was particularly striking in the anterior segment, where several missing teeth were clustered. Additionally, crown and root dysmorphisms were noted across multiple teeth, particularly in the incisors, molars, and premolars. Many teeth exhibited shortened or abnormally shaped roots, and there were marked abnormalities in the morphology of pulp chambers and root canals, indicating developmental disturbances in odontogenesis. These findings further support the diagnosis of EVC syndrome, characterized by complex anomalies in tooth formation and eruption.

The patient′s parents reported delayed dental eruption, with the first teeth erupting at approximately 2 years of age, followed by premature exfoliation shortly thereafter.

## 4. Diagnostic Assessment—New Mutation

Whole‐exome sequencing (WES) identified a novel homozygous frameshift variant in the EVC gene (NM_153717.3:c.1750delC, p.Q584Argfs∗4). No pathogenic variants were detected in EVC2, confirming EVC as the disease‐causing locus (Table 2). The variant introduces a premature stop codon, likely resulting in a truncated, nonfunctional protein.

**Table 2:** Whole‐exome sequencing results.

**Gene/transcript**	**Variant location**	**Variant**	**Chromosome position (GRCh37)**	**Zygosity**	**Related phenotypes**	**OMIM number**	**Inheritance pattern**
NM_153717	Exon 12	c.1750delC p.Q584Rfs∗4	Chr4: 5,785,463	Homozygote	Wayers acrofacial dysostosis	193530	Autosomal dominant
					Ellis–van Creveld syndrome	225500	Autosomal recessive

This mutation was absent from major population and disease databases, including ClinVar, gnomAD, and HGMD, supporting its classification as a novel variant. The EVC gene encodes a transmembrane protein with a leucine zipper domain essential for endochondral ossification and skeletal development. Pathogenic variants in EVC are associated with EVC syndrome (OMIM #225500) and, in heterozygous carriers, Weyers acrodental dysostosis (OMIM #193530).

Sanger sequencing confirmed the variant in a homozygous state in the proband and a heterozygous carrier status in both parents, consistent with an autosomal recessive inheritance pattern (Table 3). The variant was classified as pathogenic according to the American College of Medical Genetics and Genomics/Association for Molecular Pathology (ACMG/AMP) guidelines. This classification is supported by PVS1 (Pathogenic Very Strong 1), as it is a predicted loss‐of‐function frameshift variant; PM2 (Pathogenic Moderate 2), since it is absent from major population databases; and PP4 (Pathogenic Supporting 4), as the clinical phenotype is highly consistent with EVC syndrome. The segregation pattern, showing a homozygous variant in the proband and heterozygous carrier status in both parents, supports an autosomal recessive model but provides only limited evidence for PP1 (Pathogenic Supporting 1).

**Table 3:** Sanger sequencing results.

**Mutation: Gene**	**Patient**	**Father**	**Mother**
EVC: c.1750delC (p.Q584Rfs∗4)	Homozygote	Heterozygote	Heterozygote

These findings confirm the molecular diagnosis and expand the known mutational spectrum of EVC‐related EVC syndrome.

## 5. Therapeutic Intervention

A multidisciplinary treatment approach was established, involving consultation with both the pediatric dentistry and prosthodontic departments. Initial dental interventions commenced in the pediatric department at Azad University of Medical Sciences, where Teeth #55 and #65 were extracted due to their poor prognosis. Given the patient′s history of congenital heart surgery involving an anomalous venous connection, antibiotic prophylaxis was administered prior to extraction procedures (amoxicillin 50 mg/kg, oral suspension 250 mg/mL, 7.0 mL), in accordance with the American Heart Association guidelines for the prevention of infective endocarditis.

After the extractions, the patient was referred to the prosthodontics department for further treatment. During this phase, Tooth #11 erupted, and a significant diastema between Teeth #11 and #21 was noted. Composite restorations were then placed on Teeth #11 and #21 using Estelite *Σ* Quick (Tokuyama Dental Co., Tokyo, Japan).

After restorative treatment, removable partial dentures (RPDs) were constructed for both maxillary and mandibular arches to restore masticatory function, speech, and aesthetics. The prostheses were designed with Adams clasps on Teeth #16, #26, #36, and #46 to provide mechanical retention and stability. Additionally, a perforated artificial tooth (#75) was included in the design to allow for tooth eruption, providing space for future growth and preventing interference with the eruption of the permanent teeth (Figure 5).

Figure 5Prosthetic rehabilitation and restorative treatment. (a) Maxillary partial denture. (b) Intraoral occlusal view of the maxilla. (c) Mandibular partial denture with Adam′s clasps for retention and perforated artificial tooth for eruption accommodation. (d) Intraoral occlusal view of the mandible. (e) Extraoral view at the end of treatment.

**Figure:**
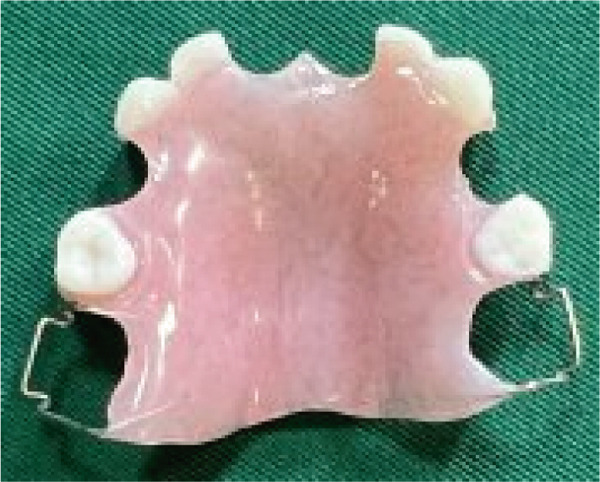
(a)

**Figure:**
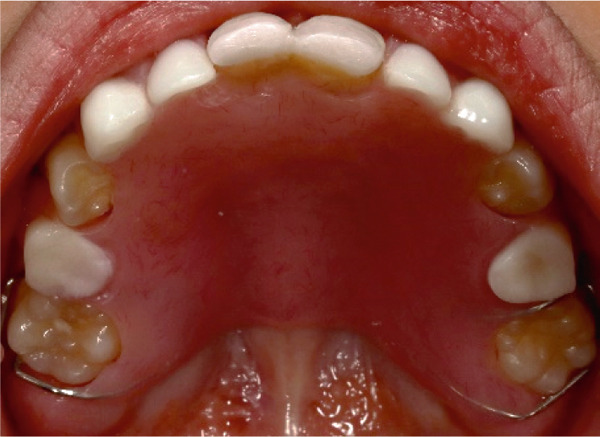
(b)

**Figure:**
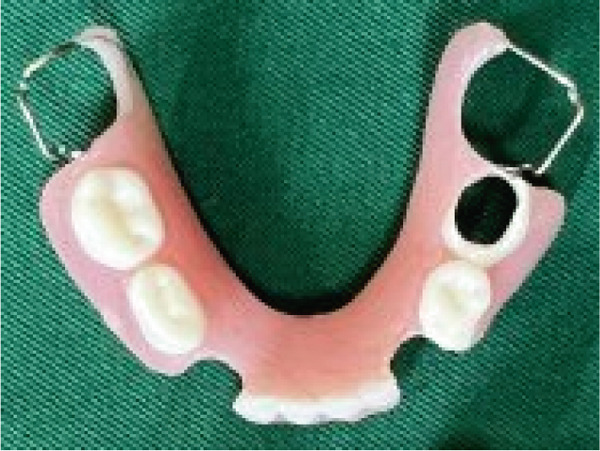
(c)

**Figure:**
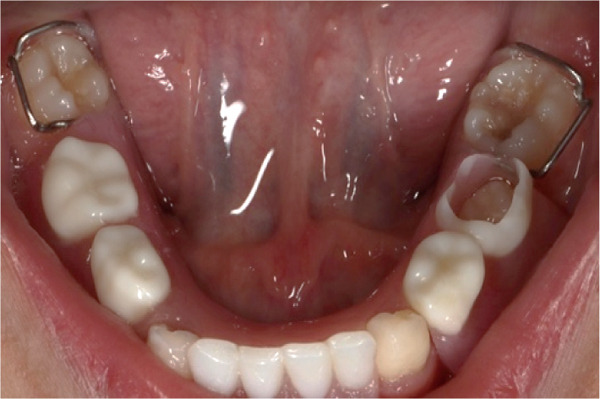
(d)

**Figure:**
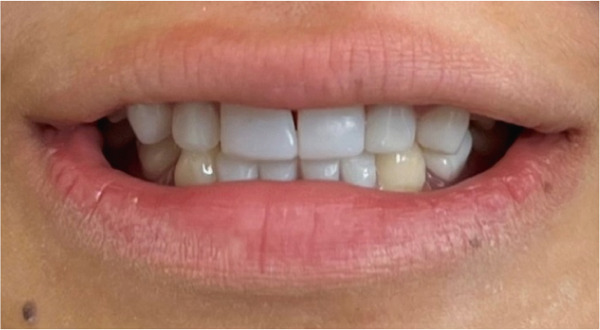
(e)

The material selected for the partial dentures was acrylic, chosen for its durability, biocompatibility, and ease of adjustment as the patient′s dental structures continue to develop. Acrylic resin is lightweight, comfortable for pediatric patients, and easily modified as the patient grows, ensuring that the dentures can be adjusted periodically without the need for a complete remake.

This treatment plan was chosen considering the patient′s age, systemic condition, and expected dental development. The prosthodontic intervention not only addressed the immediate concerns of delayed tooth eruption but also considered future growth and function, ensuring that the patient would be able to maintain oral health as he developed.

### 5.1. Justification for the Selection of a RPD

The decision to use an RPD for this pediatric patient with EVC syndrome was driven by several factors. As the patient is still in the critical stage of dental development, an RPD provides the flexibility to accommodate ongoing tooth eruption and jaw growth, unlike fixed prosthodontic options, which would require frequent remakes.

Considering the patient′s congenital heart condition, an RPD offers easier cleaning and maintenance, reducing infection risks. Its noninvasive design also allows for adjustments without impacting surrounding teeth. Additionally, RPDs are cost‐effective, easy to modify, and made from durable, biocompatible acrylic, ensuring long‐term functionality as the patient′s oral structures evolve.

## 6. Follow‐Up and Outcomes

At the 12‐month follow‐up, the patient′s partial dentures were functioning well. There were no complications reported with the prostheses, and the patient exhibited normal tooth eruption. Comparative photographs showed a significant improvement in oral aesthetics and function, while speech articulation and chewing efficiency were reported as notably improved by both the patient and caregivers. The family expressed high satisfaction with the treatment outcomes, citing better oral function, speech, and self‐confidence. During this period, the prosthesis was modified to accommodate the eruption of the permanent premolars. At the 2‐year follow‐up visit, an updated panoramic radiograph (OPG) was obtained to evaluate dental development, eruption sequence, and space availability. Based on these findings, the patient was referred for the construction of new mandibular partial dentures tailored to the current stage of dental and skeletal growth. At the time of this report, fabrication is in progress and delivery of the new prostheses is pending. The following Table 4 summarizes the patient′s major clinical events, treatments, and follow‐up.

**Table 4:** Timeline—A chronological table summarizing the onset of symptoms, diagnoses, treatments, and follow‐up would improve clarity and reproducibility.

**Age**	**Event/clinical finding**	**Intervention/outcome**
Birth	Low birth weight (2800 g, 12th percentile), short length (36 cm, 1st percentile), and macrocephaly (49 cm, 99th percentile)	Baseline anthropometrics established
Day 8	Neonatal jaundice	Phototherapy for 2–3 days, resolved without complications
4 years	Congenital cardiac anomaly identified	Cardiovascular evaluation and corrective surgery
5 years	Postaxial polydactyly	Surgical removal of supernumerary digit
5 years	Genu valgum deformity (knock knees)	Orthopedic surgical correction
11 years	Delayed tooth eruption, oligodontia, and dental malformations	Comprehensive pediatric dental evaluation, composite restorations, and initial prosthodontic rehabilitation
11–13 years	Long‐term follow‐up: Persistent short stature with relative macrocephaly; ongoing dental and orthopedic monitoring	Regular anthropometric assessment, updated panoramic radiographs (OPG) to track eruption patterns and agenesis, and construction and placement of a new partial prosthesis at 13 years to restore function and aesthetics

As the patient continues to grow, future needs will be monitored, including potential orthodontic interventions and planning for implants once growth is complete. These treatments will be tailored to the patient′s ongoing dental development and systemic condition.

## 7. Discussion

EVC syndrome is a rare autosomal recessive disorder affecting all three germ layers—primarily the ectodermal and mesodermal tissues, with occasional involvement of the endodermal layer. It is caused by mutations in the *EVC* and *EVC2* genes, both of which play crucial roles in ciliary function and cellular signaling [15, 20]. Mutations in either gene lead to similar clinical manifestations, including disproportionate short stature, polydactyly, hidrotic ectodermal dysplasia, and cardiac anomalies, which occur in 50%–60% of cases [5, 22]. Dental anomalies such as tooth agenesis, delayed eruption, and malformed crowns are also commonly observed. In this case, we identified a novel homozygous mutation (c.1750delC, p.Q584Rfs∗4) in the EVC gene, further advancing our understanding of the genetic basis of the syndrome. This novel variant, absent from population databases, contributes to the characteristic features of EVC syndrome, particularly the observed dental and skeletal abnormalities [1, 21].

This patient exhibited typical features such as short extremities, brachydactyly, and dystrophic nails [23], as well as dental anomalies including tooth agenesis, supernumerary teeth, a high‐arched palate, conical teeth, taurodontism, root and crown malformations, and multiple accessory labial frenula [16–18, 24, 25]. The absence of cardiovascular anomalies highlights the clinical variability of EVC syndrome and reinforces the importance of individualized care based on the specific manifestations present in each patient. Although other disorders like strabismus, chest wall changes, pulmonary malformations, hypospadias, epispadias, cryptorchidism, renal anomalies, dyserythropoiesis, hematologic abnormalities, perinatal myeloblastic leukemia, and central nervous system abnormalities have been reported, mental and cognitive retardation are generally not expected in this disease [22], and these additional issues were not detected in this patient. Annual physician visits are crucial to monitor and investigate potential complications. Special attention should be given to cardiovascular examinations because heart defects are the main determinant of life expectancy in these patients [4]. Since parental consanguinity is frequently documented in the literature [8, 25–27], our patient′s background of consanguineous marriage further supports the autosomal recessive inheritance pattern of the syndrome.

The differential diagnosis for EVC includes conditions such as Jeune syndrome, McKusick–Kaufman syndrome, and Weyers acrofacial dysostosis, all of which share overlapping features like polydactyly and skeletal anomalies [3, 28]. However, the presence of tooth agenesis, polydactyly, and congenital heart defects in this patient, combined with genetic confirmation, solidifies the diagnosis of EVC syndrome rather than the milder Weyers acrofacial dysostosis, which typically lacks congenital heart defects [29, 30].

The management of EVC syndrome is inherently multidisciplinary, involving orthopedic, dental, and cardiological care. Timely intervention is key in preventing complications such as malocclusion and impaired function due to delayed tooth eruption. In this case, a comprehensive treatment plan, including extractions, composite restorations, and RPDs, was implemented, with a successful 18‐month follow‐up period. This approach not only addressed the immediate dental needs but also facilitated the management of the patient′s oral development in anticipation of further eruption. Preventive measures, including oral hygiene education, dietary guidance, and professional cleanings, are critical in managing oral health and preventing dental caries in this patient [1].

Genetic counseling plays a crucial role for the family, as both parents are confirmed carriers of the pathogenic *EVC* mutation. Given the autosomal recessive inheritance pattern, there is a 25% risk of having a homozygous affected child in future pregnancies. Therefore, genetic counseling should be considered both before and during any future pregnancies. Carrier screening and genetic analysis of other family members are also recommended, particularly in consanguineous marriages, to better assess the risk of recurrence. Furthermore, prenatal diagnosis of EVC syndrome is possible by ultrasound from Week 18 of pregnancy [22].

## 8. Conclusion

The findings of this case study highlighted a novel mutation of the EVC gene c.1750delC (p.Q584Rfs∗4), emphasizing the importance of genetic analysis in the early detection of EVC syndrome, particularly for future pregnancies. The pediatric dentist plays a fundamental role in the diagnosis, follow‐up, planning, and treatment of such clinical cases. A precise treatment plan and a multidisciplinary approach were essential for providing comprehensive care, resulting in high‐quality care and satisfactory outcomes for the patient.

## Ethics Statement

Informed consent was obtained from the legal guardians of the patients included in the study.

## Consent

Written informed consent was obtained from the patient for publication of this case report and any accompanying images.

## Disclosure

None of the authors are employed by the government. They are all engaged in private practice, and all research expenses were self‐funded. All authors reviewed and approved the final manuscript.

## Conflicts of Interest

The authors declare no conflicts of interest.

## Author Contributions

M.S. contributed to conceptualization, investigation, and writing—original draft. A.P.H. contributed to investigation. H.J. contributed to investigation and writing—original draft. S.T‐H. contributed to supervision, writing—review and editing, and project administration.

## Funding

No funding was received for this manuscript.

## Data Availability

The data that support the findings of this study are available from the corresponding author upon reasonable request.
